# Antibody Response in Healthcare Workers During the SARS-CoV-2 Gamma Variant Outbreak in Manaus, Brazil

**DOI:** 10.1093/cid/ciaf318

**Published:** 2025-07-16

**Authors:** Charlene Siza, Mateusz Plucinski, Fernanda C Lessa, Evelyn Campelo, Maria Clara Padoveze, Antonio R Vieira, Gemma Parra, Guilherme Araujo, Lucia Y I Nichiata, Luciana Silva-Flannery, Kassia Lima, Aida Cristina Tapajos, Ariana Vieira, Juliette Morgan, Roberto J Freire Esteves, Barbara Marston, Cristiano Fernandes da Costa, Felipe G Naveca, Tatyana C Amorim Ramos, Pritesh Lalwani, Brazil Healthcare Personnel COVID-19 team, Kiriath Rebello, Tirza Mattos, Andresa Rocha, Aguyda Barbosa, Maria Deane, Lucas Thiago Pereira da Silva, Juliana F da Silva, Kelly M Hatfield

**Affiliations:** COVID-19 Response - International Task Force, Centers for Diseases Control and Prevention (CDC), Atlanta, Georgia, USA; Division of Healthcare Quality Promotion, Centers for Disease Control and Prevention, Atlanta, Georgia, USA; COVID-19 Response - International Task Force, Centers for Diseases Control and Prevention (CDC), Atlanta, Georgia, USA; COVID-19 Response - International Task Force, Centers for Diseases Control and Prevention (CDC), Atlanta, Georgia, USA; Division of Healthcare Quality Promotion, Centers for Disease Control and Prevention, Atlanta, Georgia, USA; Fundação de Vigilância em Saúde do Estado do Amazonas, Manaus, Amazonas, Brazil; Universidade de São Paulo, Escola de Enfermagem, São Paulo, Sao Paulo, Brazil; COVID-19 Response - International Task Force, Centers for Diseases Control and Prevention (CDC), Atlanta, Georgia, USA; Division of Healthcare Quality Promotion, Centers for Disease Control and Prevention, Atlanta, Georgia, USA; Laboratório Central do Amazonas (LACEN), Manaus, Amazonas, Brazil; Universidade de São Paulo, Escola de Enfermagem, São Paulo, Sao Paulo, Brazil; COVID-19 Response - International Task Force, Centers for Diseases Control and Prevention (CDC), Atlanta, Georgia, USA; Hospital Pronto Socorro 28 de Agosto, Manaus, Amazonas, Brazil; Hospital Pronto Socorro Platão Araújo, Manaus, Amazonas, Brazil; Hospital Pronto Socorro Platão Araújo, Manaus, Amazonas, Brazil; South America Regional Office, Centers for Disease Control and Prevention (CDC), Brasília, Federal District, Brazil; South America Regional Office, Centers for Disease Control and Prevention (CDC), Brasília, Federal District, Brazil; COVID-19 Response - International Task Force, Centers for Diseases Control and Prevention (CDC), Atlanta, Georgia, USA; Conselho de Secretarias Municipais de Saúde do Amazonas, Manaus, Amazonas, Brazil; Instituto Leônidas e Maria Deane, Fiocruz, Amazonas, Brazil; Fundação de Vigilância em Saúde do Estado do Amazonas, Manaus, Amazonas, Brazil; Instituto Leônidas e Maria Deane, Fiocruz, Amazonas, Brazil; Laboratory of Infectious Diseases and Immunology (IDI), ILMD-FIOCRUZ Amazônia/ICB-UFAM, Manaus, Amazonas, Brazil

**Keywords:** SARS-CoV-2, COVID-19, breakthrough infections, healthcare workers, antibody responses

## Abstract

**Background:**

This study aimed to evaluate severe acute respiratory syndrome coronavirus 2 (SARS-CoV-2)–specific binding and neutralizing antibody responses in healthcare workers (HCWs) who received coronavirus disease 2019 (COVID-19) vaccines, with or without postvaccination infections.

**Methods:**

We conducted a prospective, observational cohort study of HCW in 2 hospitals in Manaus, Brazil. From 31 March through 31 May 2021, HCWs had nasal swabs collected and questionnaires administered weekly for 4 visits. Nasal swabs were tested for SARS-CoV-2 by real-time reverse transcription polymerase chain reaction (rRT-PCR). Blood specimens were obtained at visits 1 and 4 unless the HCW was found to be infected. If infected, a blood specimen was collected on days 14 and 28 after symptom onset or date of positive specimen, if asymptomatic. COVID-19 vaccination cards, state immunization records, and self-reported history of previous SARS-CoV-2 infection were obtained. Fully vaccinated HCWs who tested SARS-CoV-2 rRT-PCR positive were classified as postvaccination infections.

**Results:**

A total of 771 HCWs were enrolled, with 73.7% (568/771) fully vaccinated. Anti–SARS-CoV-2 S1 immunoglobulin G and neutralizing antibody levels showed steep decay within the first 50 days after COVID-19 vaccination. HCWs with prior SARS-CoV-2 infection had slower visible decay after 50 days compared with those without prior infection. We identified 12 postvaccination infections of 16 HCWs who were SARS-CoV-2 rRT-PCR+, including 4 who also reported previous infection. Those positive for SARS-CoV-2 had lower baseline neutralizing antibody levels against Gamma and Delta variants preinfection (median log10 titers [interquartile range]: Gamma, 1.5 [3]; Delta: 0 [0.25]) compared to those who remained rRT-PCR negative (median log10 titers [interquartile range]: Gamma, 3 [2]; Delta, 1 [2]).

**Conclusions:**

Our findings highlight the importance of routine antibody surveillance, targeted boosters, and hybrid immunity in low and middle income countries. Timely booster doses for HCWs and the development of new vaccines against emerging variants can help sustain immunity and prevent workforce shortages, strengthening healthcare resilience in resource-limited settings.

## Teaser

This study, conducted in healthcare workers during a large wave of COVID-19 Gamma variant in Brazil, evaluated SARS-CoV-2–specific binding and neutralizing antibody responses over time, considering factors such as vaccination and previous infection.

Beginning in late 2020, the severe acute respiratory syndrome coronavirus 2 (SARS-CoV-2) Gamma lineage caused a large second wave of coronavirus disease 2019 (COVID-19) in Brazil. It was first reported in the city of Manaus, in Amazonas state [1]. The Gamma variant of concern (VOC) was reported to be up to 2.6 times more transmissible than wild-type SARS-CoV-2 and have a 1.2- to 1.9-fold increase in mortality risk in adults [2–4] compared to previously identified strains circulating in Brazil at the time. The increase in transmissibility continued with the Delta VOC in July 2021 and even more with the Omicron BA.1 VOC in late December 2021; however, the overall case fatality ratio was much higher during the Gamma wave (4.1) than with Delta (1.6–1.7) and Omicron (0.17) [5]. Seroprevalence studies had estimated that by October 2020, 29%–44% of the population had been infected with previous circulating strains [6]; however, statistical modeling attributed 28% of SARS-CoV-2 infections during the second wave to reinfections caused by the Gamma strain [2].

This second wave of COVID-19 caused by the Gamma VOC had significant effects on the healthcare system in Amazonas state. The number of hospitalizations increased sharply, severely straining resources [6, 7]. There were shortages in medical supplies, including oxygen, and healthcare workers (HCWs) were exhausted and overwhelmed by the number of COVID-19 patients [6, 7]. Multiple studies have described the increased risk of SARS-CoV-2 infection among HCWs because of the nature of their work [8, 9], which adds strain on the healthcare system by reducing its available workforce.

Before early 2021, there was not an approved SARS-CoV-2 vaccine available and prevention strategies had involved nonpharmaceutical interventions (NPIs). NPIs included physical distancing, masking mandates, and suspension of in-person events or mass gatherings, among other things. Manaus began relaxing physical distancing requirements in June 2020 and implemented a full-mask mandate in July 2020 [7, 10]. A study of NPIs across 27 Brazilian states from March to November 2020, including Amazonas state, concluded that although physical distancing practices increased when strict rules were in place, compliance decreased over time. Physical distancing also declined when masking mandates were implemented [10].

In January 2021, shortly after the emergence of Gamma strain, Brazil started implementation of a national vaccination strategy [11]. Two vaccines were used initially, CoronaVac and the Oxford-AstraZeneca (ChAdOx1) vaccine. CoronaVac required 2 doses given 2–4 weeks apart [12]. Phase III trials at the time across 3 countries found efficacies against symptomatic disease of 51%–84% [13, 14]. The ChAdOx1 vaccine required 2 doses [12]. Phase III trials in the United Kingdom, Brazil, and South Africa showed efficacies against symptomatic disease of 55%–81%, with a time interval between the doses of >12 weeks having a higher efficacy [15, 16].

Vaccination campaigns against SARS-CoV-2 began on 19 January 2021, using a phased approach, with prioritization of HCWs in Amazonas state given the rise of COVID-19 related to the Gamma strain [11, 17]. Of the 68 808 HCWs in Manaus, 55 584 (80.8%) and 50 029 (72.7%) had received their first and second vaccine doses, respectively, by April 2021 [17]. This study aimed to describe any postvaccination infections and the longitudinal antibody response against SARS-CoV-2 among HCWs in 2 large public hospitals in Manaus during a large wave of COVID-19.

## METHODS

### Study Design

We analyzed samples from a prospective, observational cohort study of HCW in 2 hospitals in Manaus, Amazonas State, Brazil. Enrollment and follow-up of HCW occurred from 31 March to 31 May 2021. HCWs who consented to participation had a nasal swab collected weekly regardless of symptoms for 4 consecutive weeks. Nasal swabs were tested for SARS-CoV-2 by real-time reverse transcription polymerase chain reaction (rRT-PCR). Trained study staff collected data on demographics, medical history, previous history of COVID-19, symptoms, healthcare and community exposures, and COVID-19 vaccination history using participants' COVID-19 vaccination cards or the state immunization registry. A blood specimen was obtained at enrollment (visit 1) and at the end of follow-up period (visit 4) unless the HCW was found to be infected. If infected, a blood specimen was collected at 14 and 28 days after symptom onset or date of positive specimen, if asymptomatic. HCWs with 2 documented doses of COVID-19 vaccination who tested rRT-PCR positive for SARS-CoV-2 at least 14 days after the second dose were classified as postvaccination infections. Previous infection was based on self-report of having a positive diagnosis either by laboratory test or from a physician.

### Laboratory Methods

Nasal swabs were placed in viral transport medium, stored at 2–4°C, and transported in coolers within 48 hours after collection to Laboratório Central do Amazonas for rRT-PCR for SARS-CoV-2 using AllplexTM 2019-nCoV Assay [18]. rRT-PCR results were expressed as the cycle threshold (Ct) for the gene encoding the nucleocapsid (NC) protein as previously described [19]. All samples with sufficiently low Ct values (Ct <30) were defined as a case and underwent whole-genome sequencing using COVIDSeq library preparation kit (Illumina) at Fiocruz-Amazonas, as previously described [20].

Whole blood (4 mL) was collected in ethylenediaminetetraacetic acid tubes and the plasma from these samples was tested using previously validated in-house enzyme-linked immunosorbent assay (ELISA) for SARS-CoV-2 NC immunoglobulin G (IgG) antibody titers [6], and commercially available ELISA for SARS-CoV-2 S1 IgG titers, EUROIMMUN Anti–SARS-CoV2 S1 Curve ELISA [21]. For the in-house ELISA, an anti-SARS-CoV-2 NC IgG antibody reactivity index (RI) was expressed as the ratio between optical density of the patient sample and the negative control. All samples with RI ≥1.5 were considered positive. For the commercial anti–SARS-CoV-2 S1 IgG ELISA, we used the manufacturer's cutoff of >35.2 binding antibody units per milliliter (BAU/mL) to define reactivity. After assessing serologic titer distribution in our cohort, we further classified reactive samples as low titer if the BAU/mL was from 35.3–260.03 and high titer if the BAU/mL was >260.03. This classification is consistent with data from ChAdOx1 nCoV-19 clinical trial that found a titer of ≥264 BAU/mL to be correlated with protection against SARS-CoV-2 infection [22]. Finally, we performed the SARS-CoV-2 NeutraLISA (Euroimmun) assay, a surrogate test used to determine the neutralizing capacity of anti–SARS-CoV-2 antibodies by detecting inhibition of protein interaction between the angiotensin-converting enzyme 2 receptor and the viral receptor binding domain (RBD) in the S1 domain of the spike protein. The results were expressed as a percent neutralization [23].

### Plaque Reduction Neutralization Test 90% (PRNT90)

Vaccinated HCWs with or without postvaccination infection were tested for neutralizing antibodies against the Gamma and Delta variants of SARS-CoV-2. PRNT90 was performed as previously described [24] with modifications. Briefly, the plasma samples were heat inactivated (56°C for 1 hour) and 2-fold serial dilutions were performed in maintenance medium 199 (M199). Next, diluted serum was incubated with the SARS-CoV-2 variants for 1 hour at 37°C and 5% CO_2_. For the serum-virus complexes, we used an equal amount of virus suspension containing 100 plaque forming units. Then, antibody-virus mixture was added to 24-well plates containing a confluent monolayer of Vero CCL-81 cells (ATCC, Manassas, VA, USA) and incubated for 1 hour at 37°C and 5% CO_2_. Subsequently, 1.5 mL of the overlay medium (medium 199 containing 1.5% carboxymethylcellulose and 5% fetal bovine serum) was added to each well and incubated for 4 days at 37°C and 5% CO_2_. The plates were fixed using 10% formaldehyde for 4 hours and then stained with 1% Crystal Violet for 2 hours. Plaque counting was performed under an optical microscope (Zeiss, Oberkochen, Germany). After counting the plaques, the neutralizing antibody titers were determined based on the PRNT90, considering the sera that reduced the number of plaques by 90% compared to the average of the virus control wells to be positive.

### Data Analyses

Five indicators of immune response were analyzed: IgG levels against Spike protein, IgG levels against Nucleocapsid protein, NeutraLISA percent neutralization, PRNT90 levels against SARS-CoV-2 Gamma-variant, and PRNT90 levels against SARS-CoV-2 Delta-variant. IgG levels were available and analyzed for the full cohort (N = 771 participants), while NeutraLISA and PRNT90 levels were available for a subset (N = 190).

Immune responses were plotted by time since last vaccination dose, and nonparametric locally estimated scatterplot smoothing were estimated to describe longitudinal trends in immune response decay. This analysis was performed for all participants and stratified by age category.

Pairwise correlation plots between the 5 immune response variables were created and pairwise Pearson correlation coefficients were calculated.

HCWs were grouped into 4 vaccine status categories: full, partial, unvaccinated, and unknown. Full vaccination was defined as having received the full course of doses approved for that vaccine (ie, 2 doses for CoronaVac, 2 doses for ChAdOx1) at least 14 days before sample collection. Partial vaccination was having received at least 1 but not the 2 doses of vaccine at least 14 days before sample collection.

The HCWs who were SARS-CoV-2 positive by PCR during follow-up were further analyzed. Trends in their IgG and PRNT90 levels were plotted for each HCW individually. A Gaussian model was used to assess predictors of immune response based on baseline risk factors.

Baseline IgG levels against Spike protein, Neutralisa percent neutralization, and PRNT90 levels against SARS-CoV-2 Gamma variant and Delta variant were compared between participants who remained PCR– throughout the entire study versus the 16 that had a positive PCR test during the study. Statistical significance for this comparison was measured with the Kruskal–Wallis test. All analyses were performed in R Version 4.1.2 (R Foundation, Vienna, Austria).

### Ethical Approval

This project was reviewed and approved by the institutional review board from Universidade de São Paulo (reference #4.611.867) and by the Brazilian National Ethics Committee (reference #4.858.909). This activity was reviewed by the Centers for Disease Control and Prevention (CDC), classified as nonresearch, and conducted in compliance with applicable federal laws and CDC policies. Written informed consent was obtained from all participants before enrollment.

## RESULTS

**Figure 1.**
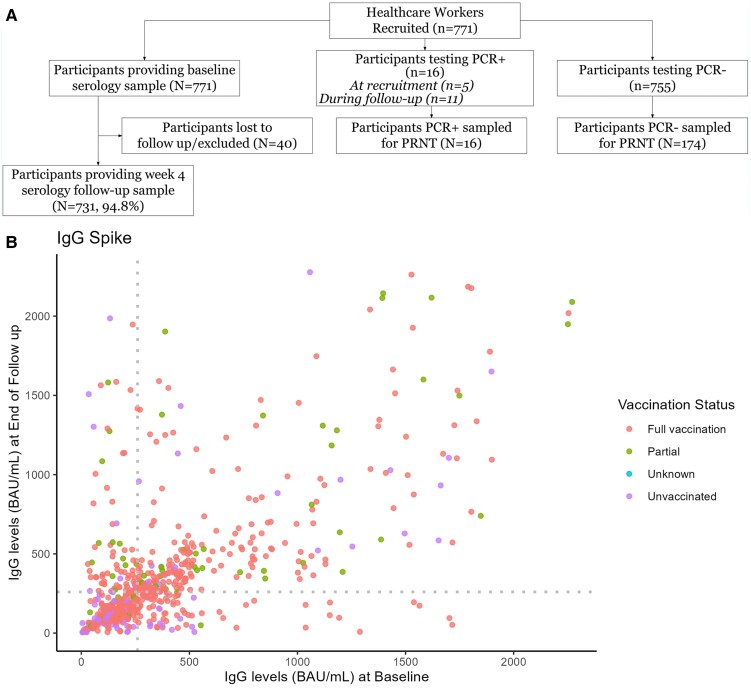
*A*, Participant enrollment, follow-up, SARS-CoV-2 PCR positive, and serological testing in the prospective study design, Manaus, Brazil, 31 March–31 May 2021. *B*, Paired anti–SARS-CoV-2 S1 IgG antibodies measured by ELISA among healthcare workers who provided blood samples at enrollment and at the end of follow-up (n = 731). Each dot represents 1 healthcare worker. Dotted vertical and horizontal lines at 260 BAU/mL mark the cutoff for high titer and indicate previously described correlates of protection against SARS-CoV-2 infection [22]. Abbreviations: BAU, binding antibody units per milliliter; ELISA, enzyme-linked immunosorbent assay; IgG, immunoglobulin G; PCR, polymerase chain reaction; SARS-CoV-2, severe acute respiratory syndrome coronavirus 2.

A total of 771 HCWs were enrolled (Figure 1*A*), with 388 from hospital A and 383 from hospital B. The HCWs were 78% (600/771) female, 14.4% (111/771) had a comorbidity, 36.1% (278/771) had been physically active in the previous 30 days, and the mean age was 40 years (range: 18–74 years). At enrollment, 73.7% (568/771) were fully vaccinated, 9.2% (71/771) were partially vaccinated, 16.9% (130/771) were unvaccinated, and for 0.3% (2/771) the status was unknown. Of those fully or partially vaccinated, 98% (623/639) received CoronaVac and 2% (16/639) received ChAdOx1. Throughout the study period, there were 16 HCWs who were SARS-CoV-2 PCR positive (2.1%), 12 of whom had been fully vaccinated. Among the 16 who were PCR positive, 6 reported previous infection (4 fully vaccinated, 1 partially vaccinated, and 1 unvaccinated). Whole genome sequencing was performed successfully on 6 samples, and sequences were genotyped as Gamma VOC for all 6 samples. The other 10 samples had a cycle threshold above 30 and were unable to be fully sequenced (data not shown).

### Description of Trends in Immune Response

**Figure 2.**
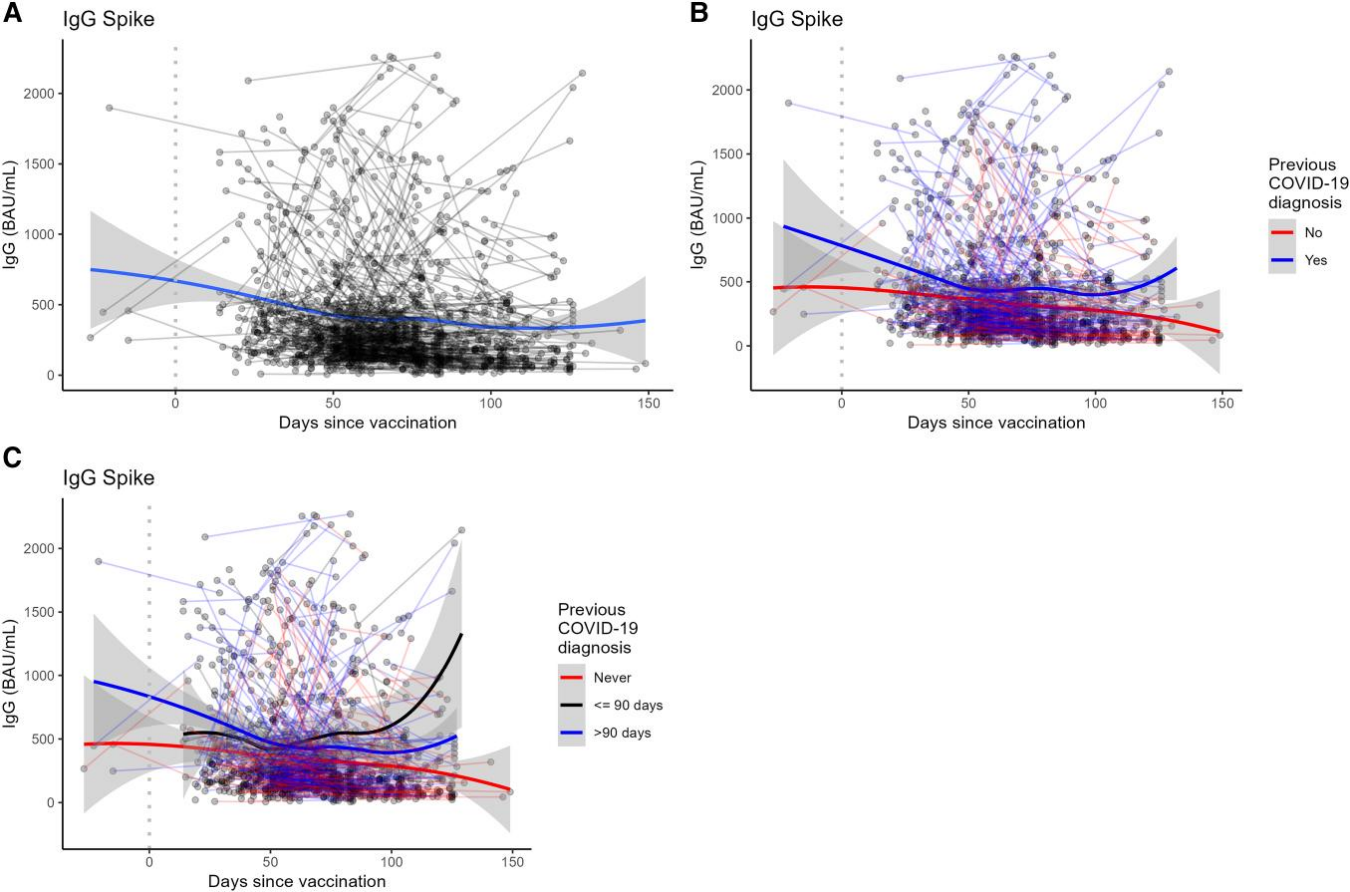
Trends in anti–SARS-CoV-2 S1 IgG antibody responses measured in vaccinated healthcare workers (n = 589) followed longitudinally in Manaus, Brazil, 31 March–31 May 2021. Lines connect samples from the same individual at different time points. Excludes 16 participants who were SARS-CoV-2 PCR+ during the study. Dotted vertical line denotes day of vaccination. *A*, S1 IgG response by days since vaccination without stratification by previous COVID-19 diagnosis. *B*, S1 IgG response by days since vaccination while stratifying by those who reported a previous COVID-19 diagnosis and those who did not. *C*, S1 IgG response by days since vaccination with further stratification by those who reported a previous COVID-19 diagnosis in the last 90 days, those who reported a previous diagnosis more than 90 days in the past, and those who did not report a previous diagnosis. Abbreviations: PCR, polymerase chain reaction; SARS-CoV-2, severe acute respiratory syndrome coronavirus 2.

**Figure 3.**
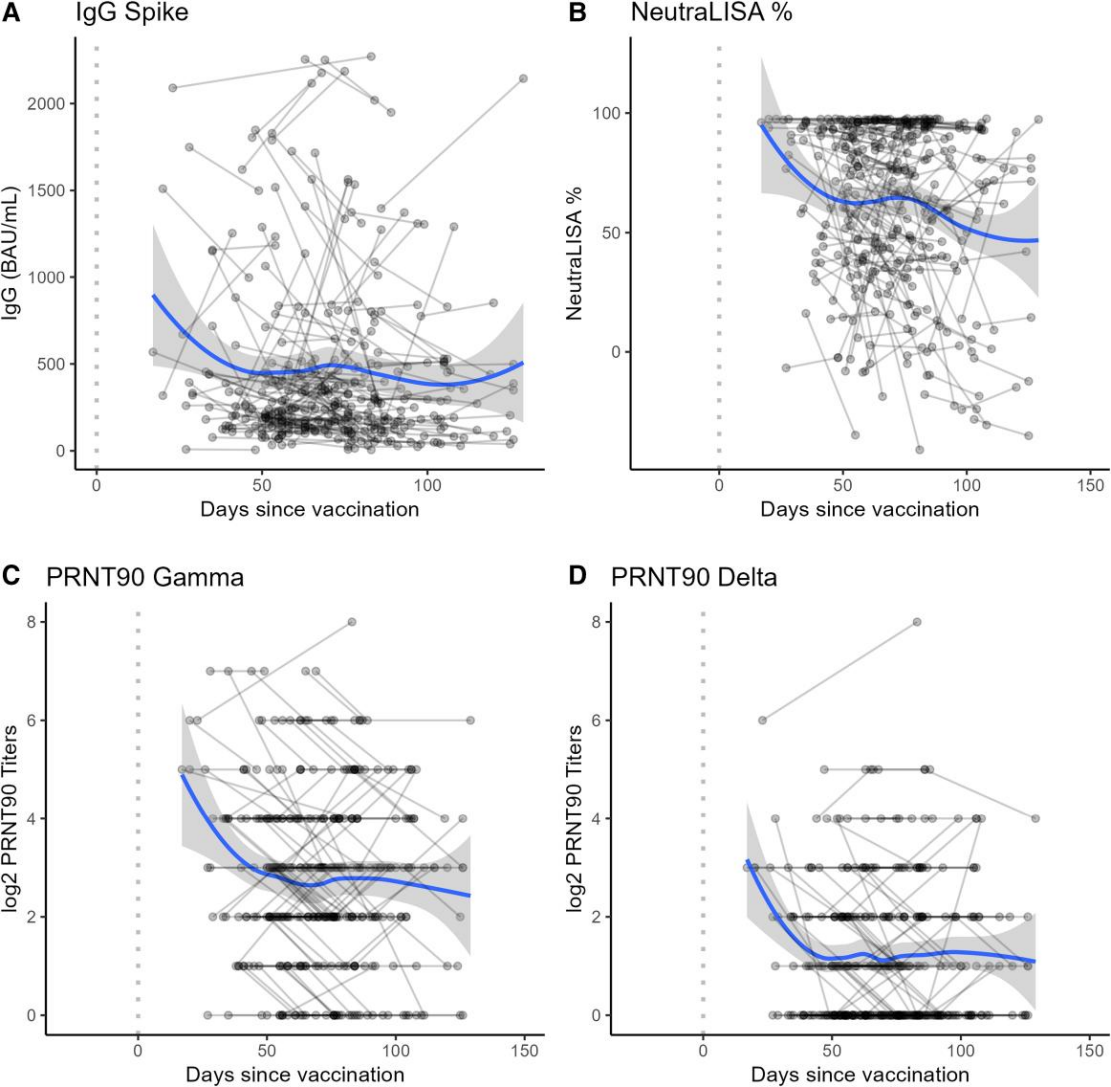
Trends in immune responses against SARS-CoV-2 measured in subset of vaccinated healthcare workers with available neutralization data (n = 174), Manaus, Brazil, 31 March–31 May 2021. Lines connect samples from the same individual at different time points. Excludes 16 participants who were SARS-CoV-2 PCR+ during the study. Dotted vertical line denotes day of vaccination. Nonparametric locally estimated scatterplot smoothing (LOESS) lines with confidence intervals were estimated to describe longitudinal trends in immune response decay. *A*, S1 IgG antibody response by days since vaccination. *B*, Percent antibody neutralization based on NeutraLISA test by days since vaccination. *C*, PRNT90 Gamma titers (with log2 transformation) by days since vaccination. *D*, PRNT90 Delta titers (with log2 tranformation) by days since vaccination. Abbreviations: PCR, polymerase chain reaction; SARS-CoV-2, severe acute respiratory syndrome coronavirus 2.

Baseline S1 IgG levels at enrollment and end of follow-up are described in Figure 1*B*. During follow-up, 53.6% (392/731) of the participants remained below 260 BAU/mL anti-SARS-CoV-2 S1 IgG antibody titers (Figure 1*B*). Anti–SARS-CoV-2 S1 IgG antibody titers declined with time since the most recent vaccine dose for IgG levels against the Spike and Nucleocapsid proteins (Figure 2, Supplementary Figure 1), for NeutraLISA neutralization percentage, and for PRNT90 titers against Gamma and Delta variants (Figure 3). The steepest declines for S1 IgG and neutralizing antibody responses occurred in the first 50 days following their most recent vaccine dose, with a plateau between 50 and 100 days postdose (Figure 3). Responses were generally higher in individuals with reported previous COVID-19 (Figure 2*B*, Supplementary Table 1). Amongst individuals with previous COVID-19, an infection in the previous 90 days resulted in the highest levels of immune response (Figure 2*C*). Trends in the decay of the immune response did not vary by age except for the age group >60 years (Supplementary Figure 2).

The 5 measures of immune response we evaluated had statistically significant positive correlations between each other (Supplementary Figure 3). The highest correlation was between PRNT90 titers to Gamma and Delta variants, followed by PRNT90 titers to Gamma variant and Spike IgG levels. PRNT90 titers to Delta were largely not detectable for low to moderate IgG responses, only appearing for individuals with high IgG or NeutraLISA responses (bottom row of Supplementary Figure 3).

Median PRNT90 titers against Delta were 4 times lower (interquartile range [IQR]: 2) compared to median PRNT90 titers against Gamma in the same sample (Supplementary Figure 4). Of the samples with non-zero PRNT90 titers against Gamma, 37.9% (131/346) had undetectable PRNT90 activity against Delta. Conversely, 100.0% (215/215) of samples with observed, non-zero PRNT90 titers against Delta also had detectable PRNT titers against Gamma.

### Immune Response in Participants Testing PCR+ for SARS-CoV-2

A total of 16 participants had positive SARS-CoV-2 rRT-PCR+ results at 1 point during recruitment or follow-up, with 12 of these being fully vaccinated, 1 partially vaccinated, and 3 unvaccinated (Supplementary Figure 5). When considering IgG antibody response to spike protein, only 3 showed increase following infection. When considering PRNT titers against the Gamma variant, 9 of the 16 showed increases following infection (Supplementary Figure 5).

**Figure 4.**
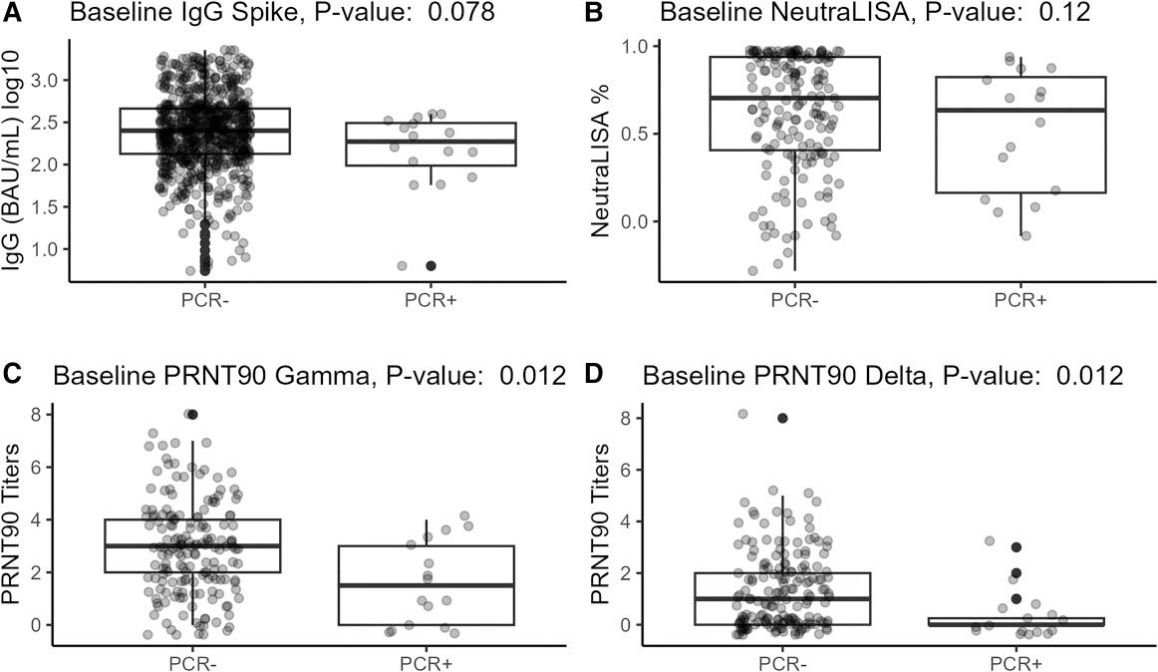
Baseline antibody responses measured among healthcare workers, Manaus, Brazil, 31 March–31 May 2021. Active follow-up and screening were performed among HCW during the study period. Participants with a positive SARS-CoV-2 PCR test during the study period are compared to PCR-negative participants. *P* value is from the Kruskal–Wallis Test for difference between the groups. *A*, Baseline S1 IgG response (n = 771) among participants with a positive SARS-CoV-2 PCR test and those with a negative test during the study period. *B*, Baseline NeutraLISA percent neutralization (n = 190) among participants with a positive SARS-CoV-2 PCR test and those with a negative test during the study period. *C*, Baseline PRNT90 Gamma response (n = 190) among participants with a positive SARS-CoV-2 PCR test and those with a negative test during the study period. *D*, Baseline PRNT90 Delta response (n = 190) among participants with a positive SARS-CoV-2 PCR test and those with a negative test during the study period. Abbreviations: HCW, healthcare worker; IgG, immunoglobulin G; PRNT90, Plaque Reduction Neutralization Test 90%; SARS-CoV-2, severe acute respiratory syndrome coronavirus 2.

The only immune response level at baseline associated with testing rRT-PCR + during the follow-up was PRNT baseline titers (Figure 4). Baseline titers were lower in those who tested rRT-PCR + (median Log10 titers [IQR]: Gamma, 1.5 [3]; Delta, 0 [0.25]) during follow-up versus those who were rRT-PCR– throughout the study period (median Log10 titers [IQR]: Gamma, 3 [2]; Delta, 1 [2]) (Figures 4*C* and 4*D*).

## DISCUSSION

This study assessed SARS-CoV-2–specific binding and neutralizing antibody responses in HCWs who received COVID-19 vaccines with or without postvaccination infection. We identified Gamma variant infection among the HCWs both at recruitment and during follow-up. Previously infected vaccinated HCWs showed greater increases in both S-RBD and N-specific antibody responses. Individuals with positive SARS-CoV-2 rRT-PCR during follow-up had lower Gamma- and Delta-specific neutralizing antibodies at baseline compared to those who were negative, regardless of vaccination status at baseline. Gamma and Delta variants have shown an increased ability to escape neutralization and our results are consistent with these observations [25, 26]. While not evaluated for decay rate, we observed a sharp decline in neutralizing antibodies 2 months after last vaccine dose. Among individuals who reported a previous infection, while the initial decay appears to be faster, it plateaus slightly more than those without previous infection. Overall, monitoring of variant-specific antibody levels can detect vulnerable groups that may need additional or more frequent vaccine doses.

Identifying immune correlates of protection (or lack thereof) from SARS-CoV-2 is critical to predicting how the expected antibody decay will affect clinical outcomes. The assumption that the presence of neutralizing antibodies would correlate with protection from re-infection with SARS-CoV-2 has been supported by studies comparing the incidence of infection between seropositive and seronegative persons. Variant-specific neutralizing antibody level are highly predictive of immune protection when comparing population values from vaccine efficacy and immunogenicity trials [27, 28].

The visual decline in antibody levels after the second COVID-19 vaccine dose exhibited a similar trend to those of natural SARS-CoV-2 infection. Importantly, the anti-SARS CoV-2 S1 IgG, NeutraLISA, and Gamma and Delta PRNT responses all showed decreases with time since last vaccine dose. This relationship was most pronounced with PRNT levels against Gamma and Delta variant declining 1.3- to 1.6-fold within 25 days after the last vaccine dose (data not shown). Intense transmission had been observed in Manaus during the first wave of SARS-CoV-2 (before emergence of Gamma) likely related to noncompliance with nonpharmaceutical interventions measures (eg, not wearing a mask during contact, not avoiding close contact, nonremote working in Manaus) [29]. Then in late December 2020, a significant surge of postvaccination infections was observed in the city of Manaus with the appearance of the Gamma variant [20]. The introduction of CoronaVac vaccination in HCW and the general population resulted in a significant reduction in hospitalization among postvaccination infections in a test-negative analysis [17].

At enrollment, data on previous COVID-19 were self-reported, since Manaus city lacked widespread rRT-PCR testing in the early part of the pandemic, and individuals had mostly been diagnosed clinically or by point-of-care COVID-19 serological tests. Additionally, because HCWs were prioritized for vaccination as soon as the vaccine became available in the country, a large proportion of our study cohort was already vaccinated at recruitment, and it was not possible to ascertain the self-reported previous infection. However, the hybrid immunity arising from both previous COVID-19 and vaccination could significantly enhance neutralizing antibodies compared to individuals with only vaccination. A previous case-control study conducted in Manaus found no major differences in anti-RBD antibodies between postvaccination and CoronaVac vaccinated individuals; however, they not only observed higher levels of anti-RBD and neutralizing antibodies but also a slower decline in humoral response postvaccination among individuals with hybrid immunity [30].

Among some vaccinated HCWs with a Gamma variant postvaccination infection, we also observed an increased neutralization activity. The breadth of neutralizing antibodies has been shown to increase after a postvaccination infection [31, 32]. SARS-CoV-2 viral load during natural and postvaccination infection is has been shown to correlate with the magnitude of the immune response and disease severity as well [33–35]. Higher antibody levels correlate with a better neutralization capacity and may provide better or longer protection against severe SARS-CoV-2 infections [36].

Despite the valuable insights provided by our study, there are some limitations that should be acknowledged. First, our study was conducted among HCWs in specific hospital settings in Manaus, Amazon State, Brazil, which may limit the generalizability of our findings to broader general populations. Additionally, our study focused on a relatively short follow-up period from 31 March to 31 May 2021, which may not capture longer-term changes in immune responses following vaccination. Long-term follow-up studies are needed to assess the durability of vaccine-induced immunity and the potential need for updated doses over time.

Furthermore, our study assessed immune responses based on laboratory measurements of IgG levels against specific viral proteins and neutralization PRNT90 titers against certain variants. Although these measurements provide valuable information about vaccine-induced immunity, they may not fully capture the breadth and complexity of the immune response to SARS-CoV-2 infection or vaccination. Future studies that include measurement of T-cell response after vaccination could provide more insight into the persistence of immune response and correlates of protection. Another limitation of our study is the potential for selection bias, as participation in the study was voluntary and may have been influenced by factors such as individuals' perceptions of COVID-19 risk.

Despite these limitations, our study contributes important insights into the dynamics of immune responses following COVID-19 vaccination among HCWs in Brazil. Our data show that infections occurred in previously vaccinated individuals in our study population but our analysis does not determine whether the incidence was lower compared to unvaccinated individuals. Although vaccine-induced humoral immunity did not prevent postvaccination infections by the Gamma variant, it may have played a role in modulating disease severity, a hypothesis that warrants further investigation. Recent vaccine dose or postvaccination infection increases the neutralizing capacity against the infecting variant. Additional updated vaccine doses would further boost humoral immunity rather than T-cell–mediated immunity, which remains stable for longer periods after COVID-19 vaccination and provides protection by providing cross-recognition between variants, both of which are likely essential in preventing severe COVID-19 disease [37–39]. In this context, our results on infection-induced antibody responses suggest that hybrid immunity should be considered in vaccination policy and in predicting the impact of SARS-CoV-2 surges, and that the development of new vaccines against emerging variants should be continued to reduce the number of postvaccination and severe infections. Additionally, given the constant risk of emerging variants, our findings highlight the importance of antibody surveillance and targeted booster campaigns in LMICs. Ensuring timely updated booster doses for HCWs can help maintain immunity levels, reducing the likelihood of severe infections and workforce shortages during outbreaks. Such strategies are crucial for sustaining healthcare system capacity, particularly in resource-limited settings where disruptions can have severe consequences.

## Supplementary Material

ciaf318_Supplementary_Data

## Supplementary Data

Supplementary materials are available at *Clinical Infectious Diseases* online. Consisting of data provided by the authors to benefit the reader, the posted materials are not copyedited and are the sole responsibility of the authors, so questions or comments should be addressed to the corresponding author.
