# Supplementary material for: Antibody Response in Healthcare Workers During the SARS-CoV-2 Gamma Variant Outbreak in Manaus, Brazil
Source: Clin Infect Dis. 2025 Jul 16;81(2):239–47. doi: 10.1093/cid/ciaf318 (PMC12448572; doi:10.1093/cid/ciaf318)
Supplement: ciaf318_Supplementary_Data [file ciaf318_supplementary_data.zip › Supplementary Material_Figure legends and Table S1.docx]

**SUPPLEMENTARY MATERIAL**

**Antibody response to SARS-CoV-2 in healthcare workers during SARS-CoV-2 Gamma variant outbreak in Manaus, Brazil**

**Authors:** Charlene Siza^1,2^Mateusz Plucinski^1^, Fernanda C. Lessa^1,2^, Evelyn Campelo^3^, Maria Clara Padoveze^4^, Antonio R. Vieira^1^, Gemma Parra^2^, Guilherme Araujo^5^, Lucia I. Nichiata^4^, Luciana Silva-Flannery^1^, Kassia Lima^6^, Aida Cristina Tapajos^7^, Ariana Vieira^7^, Juliette Morgan^8^, Roberto J. Freire Esteves^8^, Barbara Marston^1^, Cristiano Fernandes da Costa^9^, Felipe G. Naveca^10^, Tatyana C. Amorim Ramos^3^, Pritesh Lalwani^10, 11^, *Brazil Healthcare Personnel COVID-19 team

# *Brazil Healthcare Personnel COVID-19 team are listed in the Acknowledgments

**
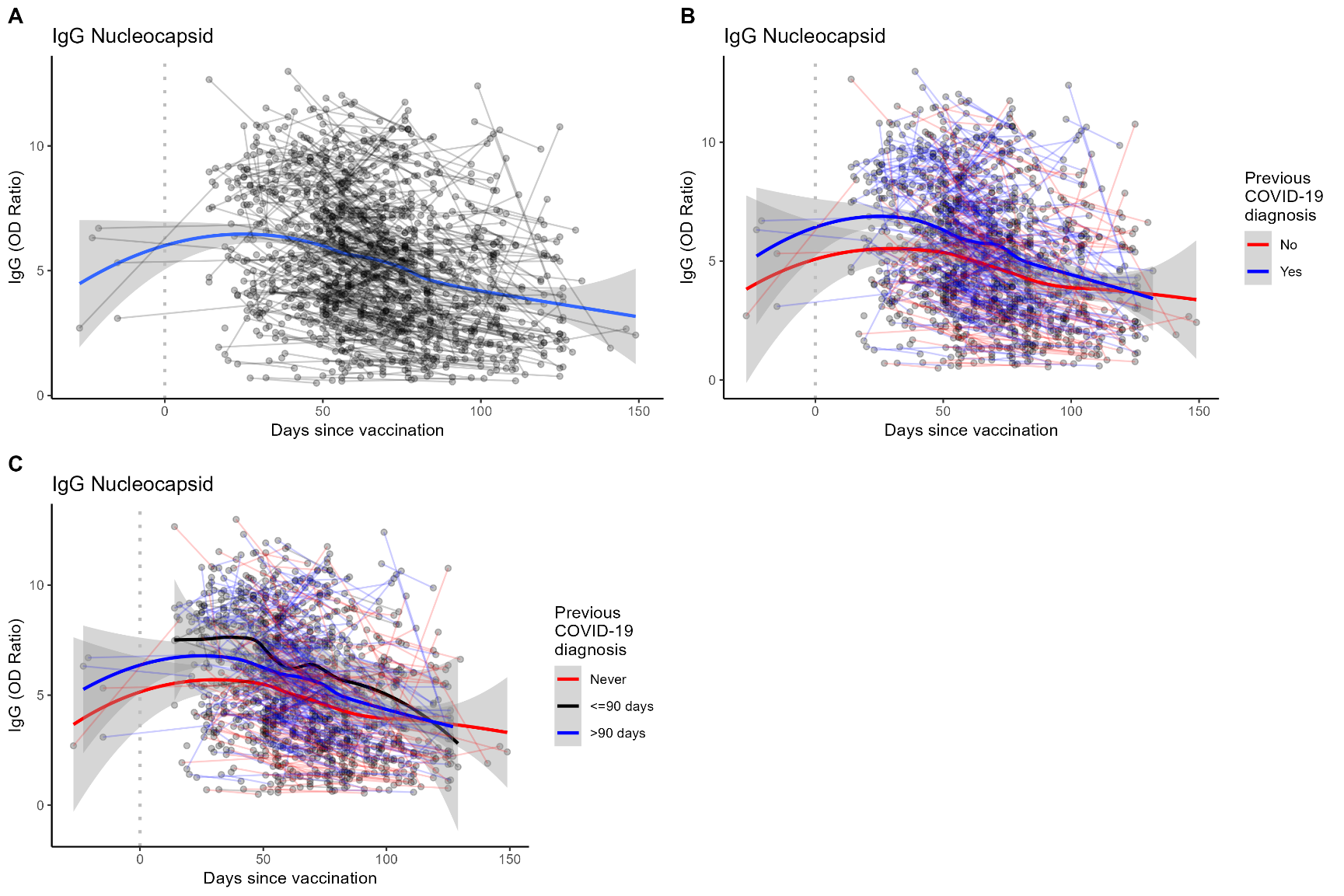
Figure S1**. Trends in immune responses to the SARS-CoV-2 nucleocapsid protein measured in healthcare workers followed longitudinally in Manaus, Brazil, March 31–May 31, 2021. (n=755) Lines connect samples from the same individual at different time points. Excludes 16 participants with PCR+ during the study. A, IgG nucleocapsid response by days since vaccination, with no stratification. B, IgG nucleocapsid response by days since vaccination, stratified by presence or absence of previous COVID-19 diagnosis. C, IgG nucleocapsid response by days since vaccination, stratified by previous COVID-19 diagnosis in the last 90 days, greater than 90 days previously, or no previous diagnosis.

**Figure S2**. Age-stratified trends in immune response measured in healthcare workers followed longitudinally in Manaus, Brazil, March 31–May 31, 2021. Lines connect samples from the same individual at different time points. Excludes 16 participants PCR+ during the study. A, S1 IgG response by days since vaccination stratified by age group. B, IgG nucleocapsid response by days since vaccination stratified by age group. C, NeutraLISA percent neutralization by days since vaccination stratified by age group. D, PRNT90 Gamma response by days since vaccination stratified by age group. E, PRNT90 Delta response by days since vaccination stratified by age group.


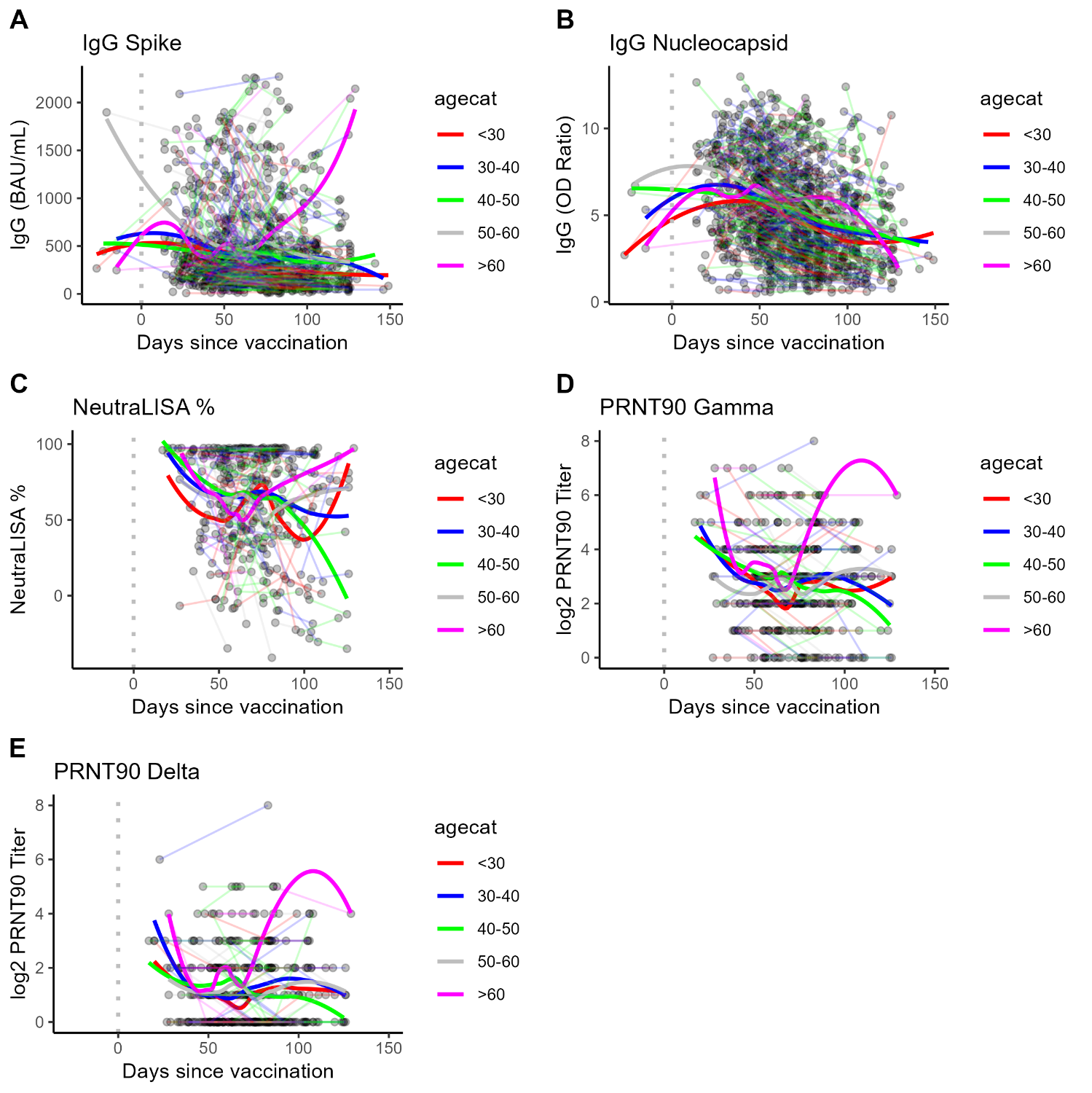


**Figure S3**. Pairwise Pearson correlation between different measures of immune response to SARS-CoV-2 in healthcare workers in Manaus, Brazil, March 31–May 31, 2021. Values with the symbol *** indicate a p-value of <0.001.

**
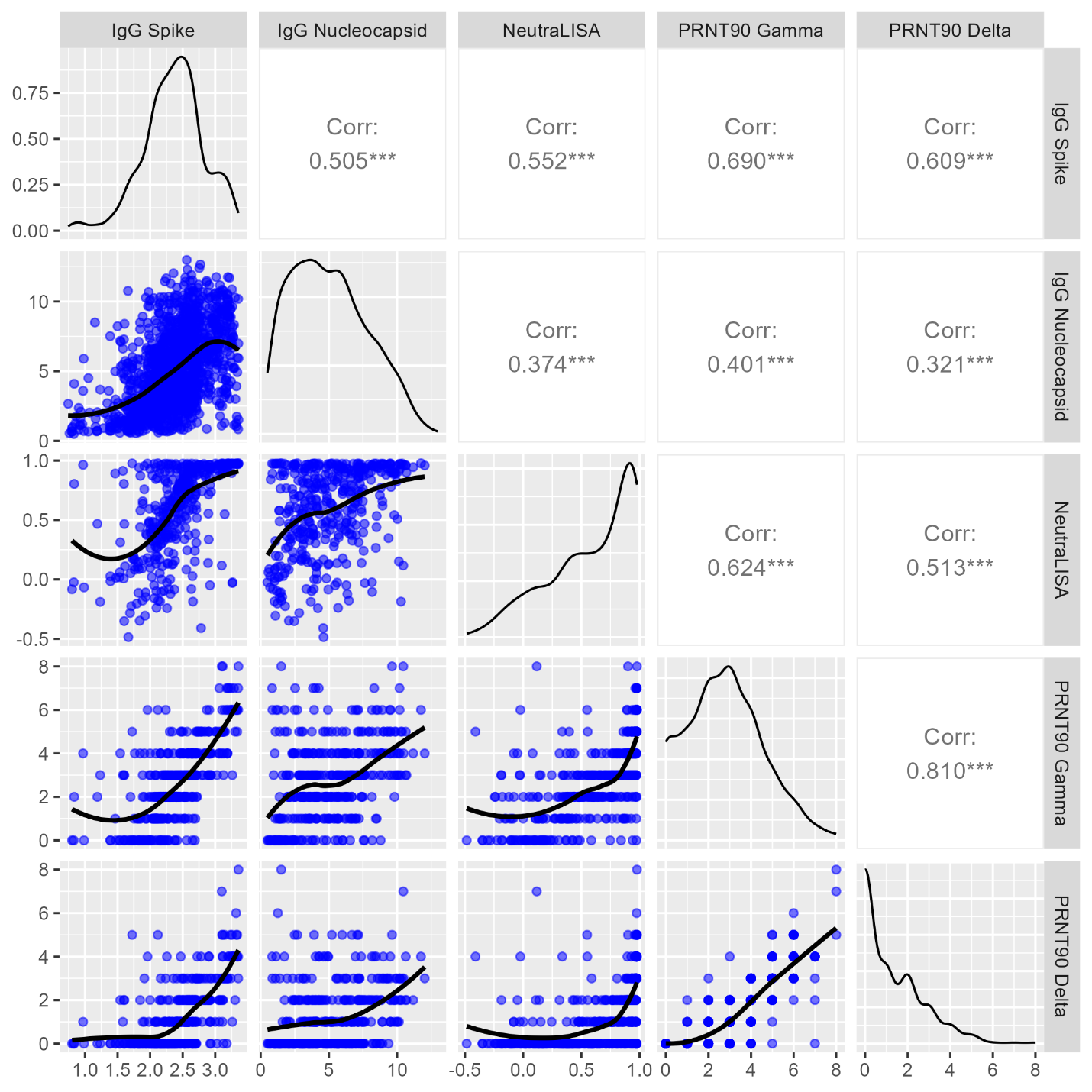
**

**Figure S4**. Plaque reduction neutralization test (PRNT) titers against Delta and Gamma SARS-CoV-2 measured in healthcare workers in Manaus, Brazil, March 31–May 31, 2021. Lines connect titer observations from same sample.


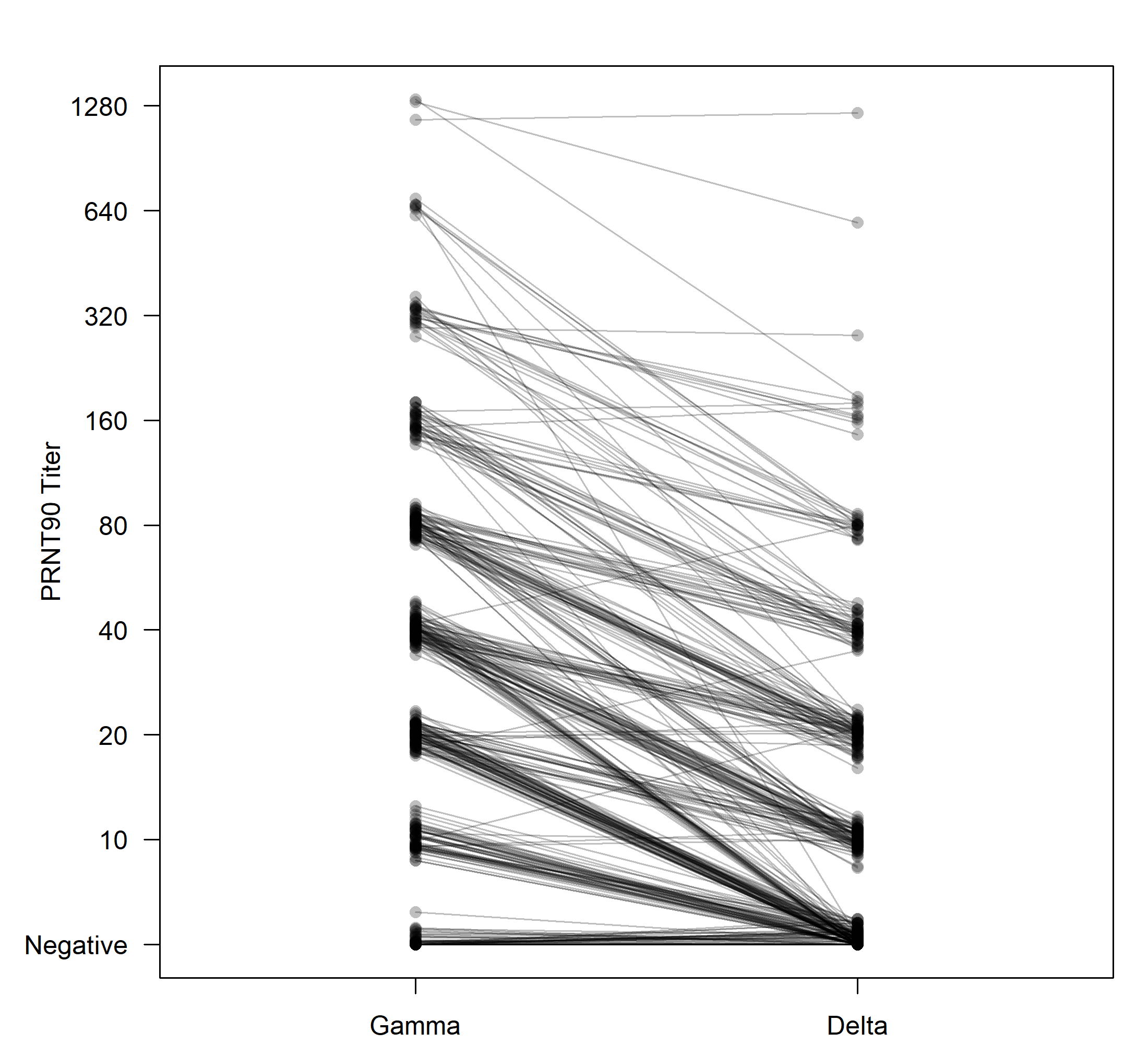


**
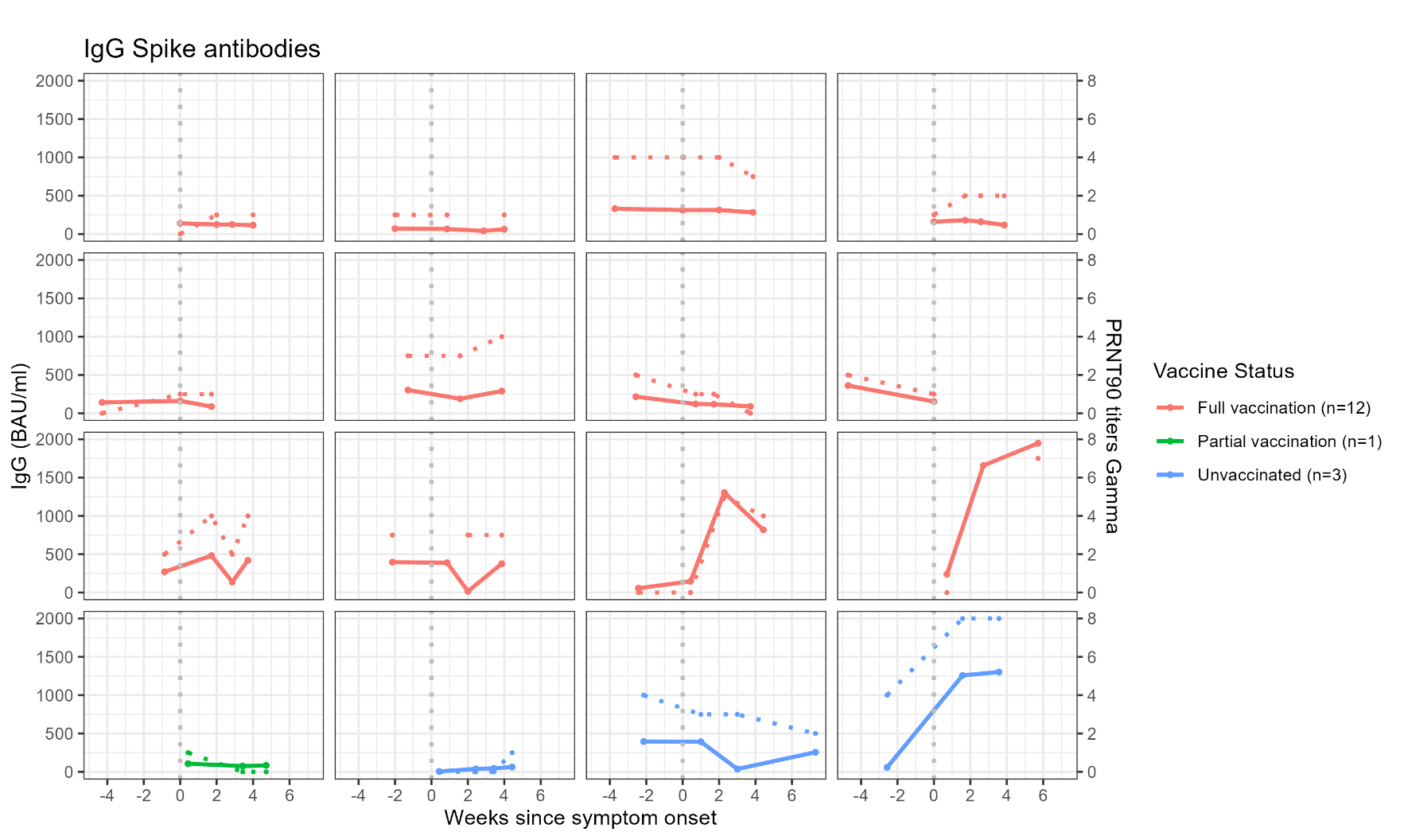
Figure S5**. Immune responses in 16 RT-PCR positive participants by time since symptom onset or positive test observed during longitudinal follow up of healthcare workers in Manaus, Brazil, March 31–May 31, 2021. Solid line denotes anti-SARS-CoV-2 S1 IgG levels and dotted line denotes PRNT titers to Gamma. The 13 fully or partially vaccinated HCWs were vaccinated with CoronaVac.

| **Table S1**. Factors associated with testing PCR+ in vaccinated healthcare workers followed longitudinally in Manaus, Brazil, March 31–May 31, 2021 | | |
| --- | --- | --- |
|  | **OR** | **95% CI** |
| Age |  |  |
| < 30 years | Ref |  |
| 30-40 years | 0.52 | 0.041, 5.6 |
| 40-50 years | 1.3 | 0.22, 10 |
| 50-60 years | 0.76 | 0.093, 7.3 |
| >60 years | 1 | 0.036, 16 |
| Sex |  |  |
| Female | Ref |  |
| Male | 1.4 | 0.25, 5.9 |
| Physically active |  |  |
| No | Ref |  |
| Yes | 1.7 | 0.43, 6.3 |
| Baseline Vaccine Status |  |  |
| Partial | Ref |  |
| Full | 0.18 | 0.009, 6.3 |
| Chronic medical condition |  |  |
| No | Ref |  |
| Yes | 3.2 | 0.73, 13 |
| Previous COVID-19 diagnosis |  |  |
| No | Ref |  |
| Yes | 0.86 | 0.22, 3.3 |
| Baseline IgG Spike | 1.3 | 0.15, 13 |
| Baseline IgG Nucleocapsid | 1 | 0.72, 1.4 |
| Baseline NeutraLISA | 5.3 | 0.42, 87 |
| Baseline PRNT Gamma | 0.42 | 0.21, 0.76 |
