# Supplementary figures and images for: Antibody Response in Healthcare Workers During the SARS-CoV-2 Gamma Variant Outbreak in Manaus, Brazil
Source: Clin Infect Dis. 2025 Jul 16;81(2):239–47. doi: 10.1093/cid/ciaf318 (PMC12448572; doi:10.1093/cid/ciaf318)

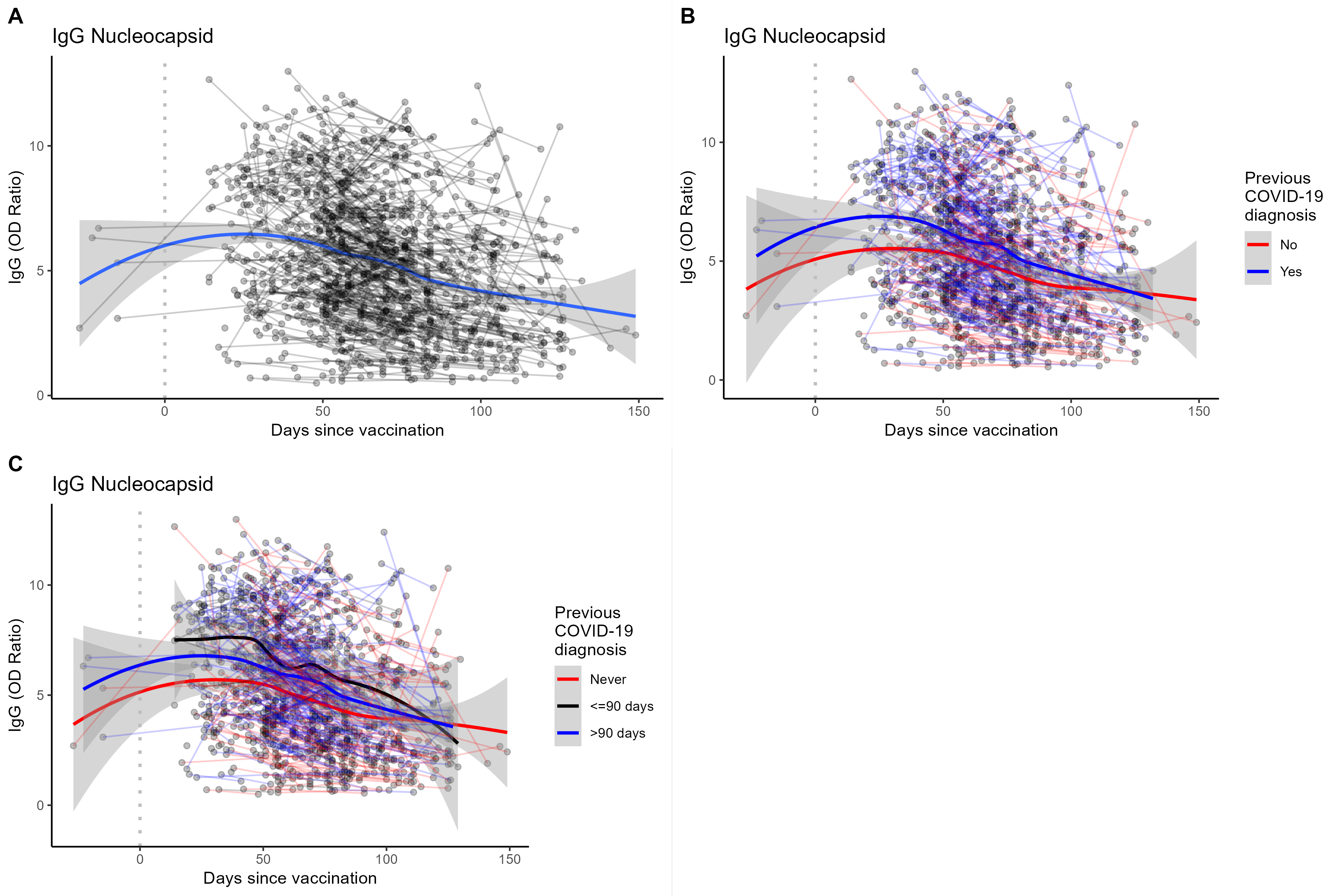

Supplement: ciaf318_Supplementary_Data [file ciaf318_supplementary_data.zip › Figure S1.tiff]

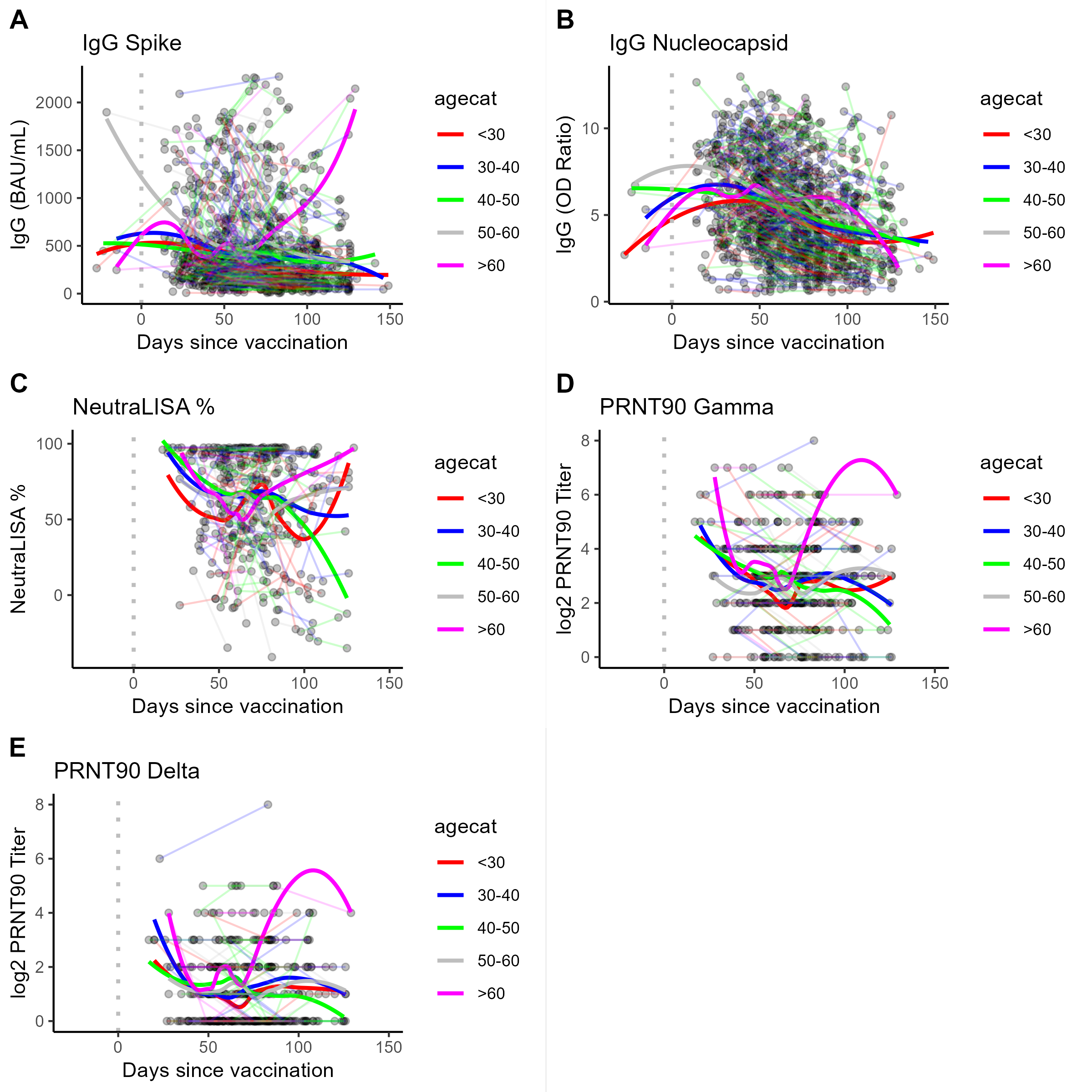

Supplement: ciaf318_Supplementary_Data [file ciaf318_supplementary_data.zip › Figure S2.tiff]

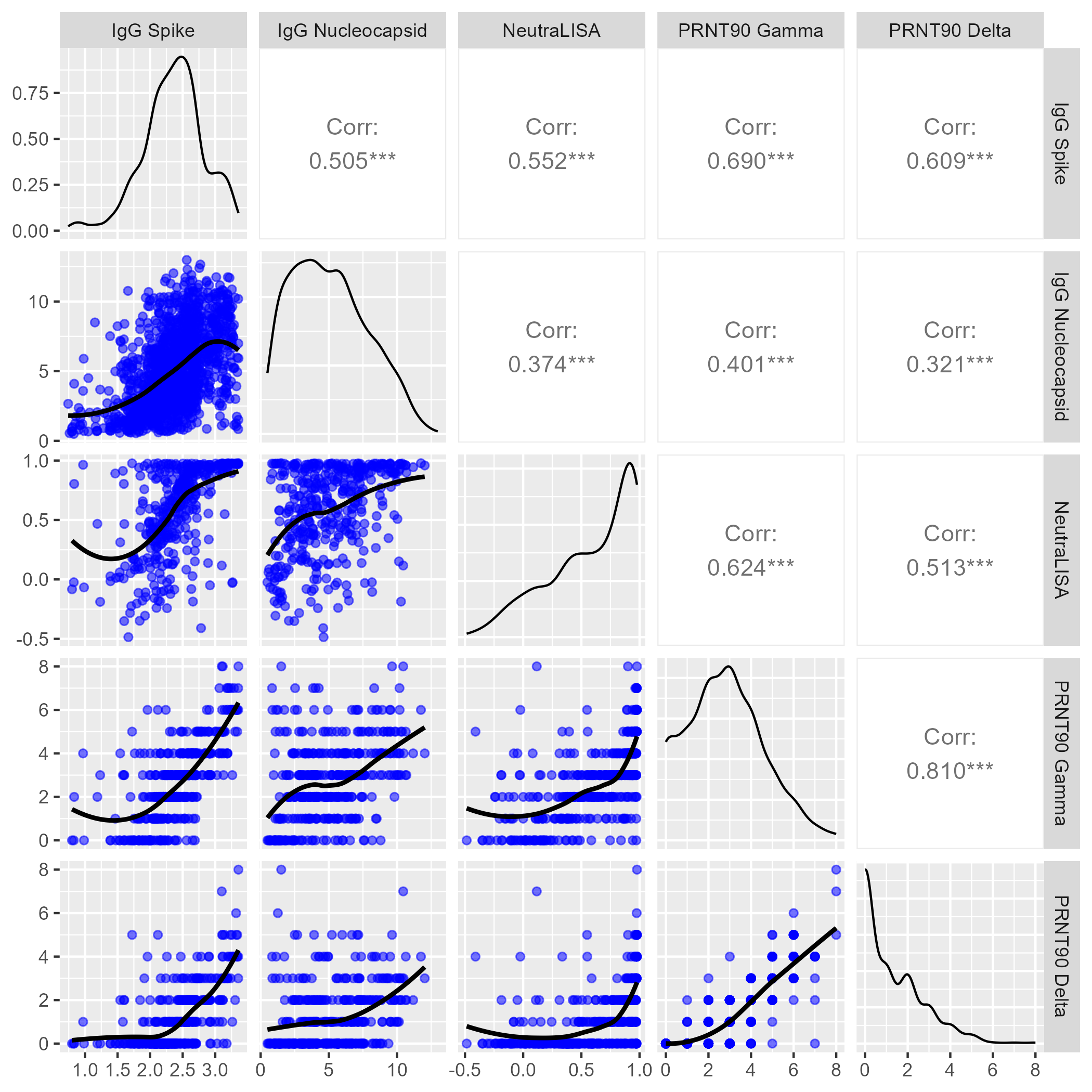

Supplement: ciaf318_Supplementary_Data [file ciaf318_supplementary_data.zip › Figure S3.tiff]

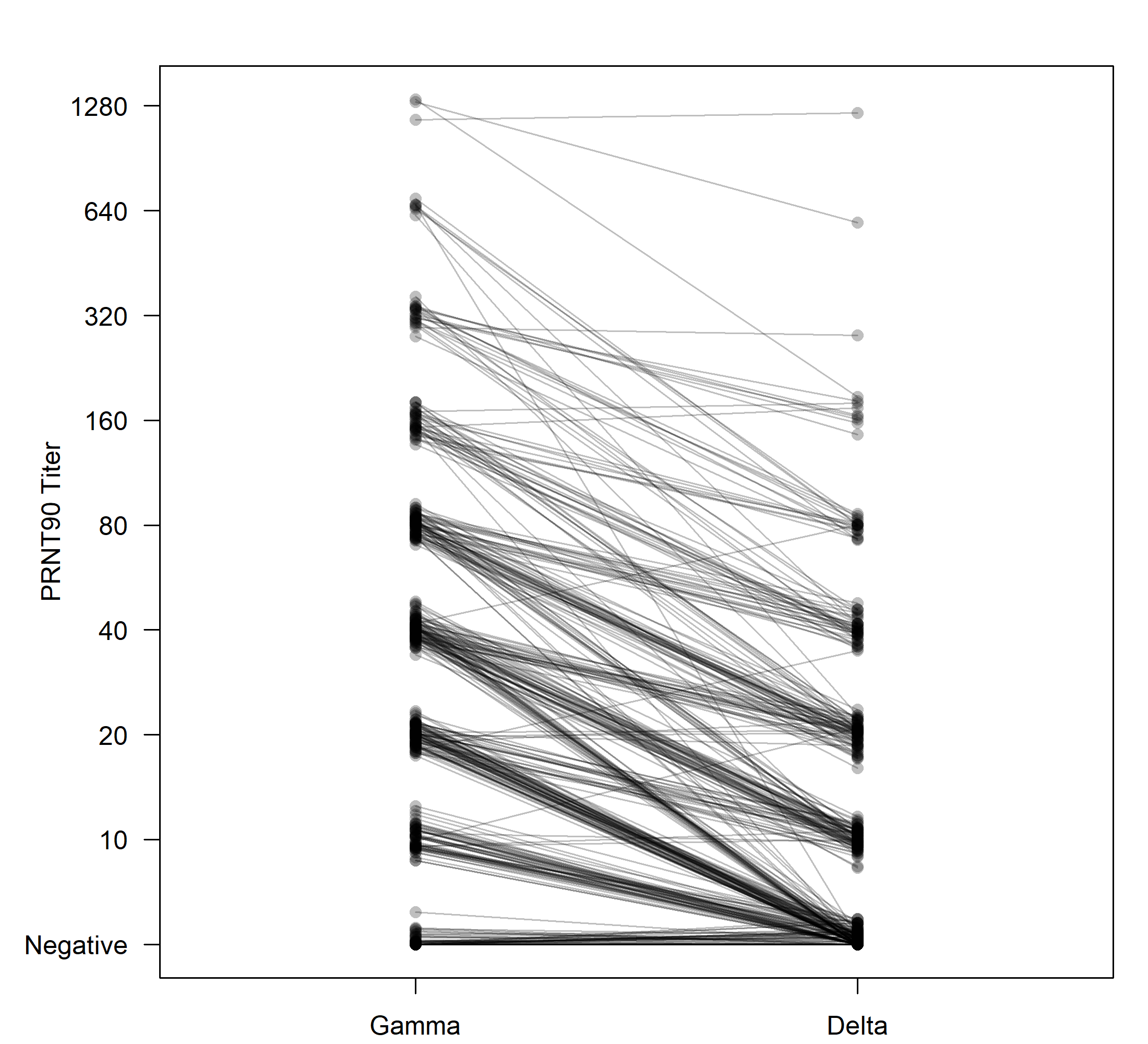

Supplement: ciaf318_Supplementary_Data [file ciaf318_supplementary_data.zip › Figure S4.tiff]

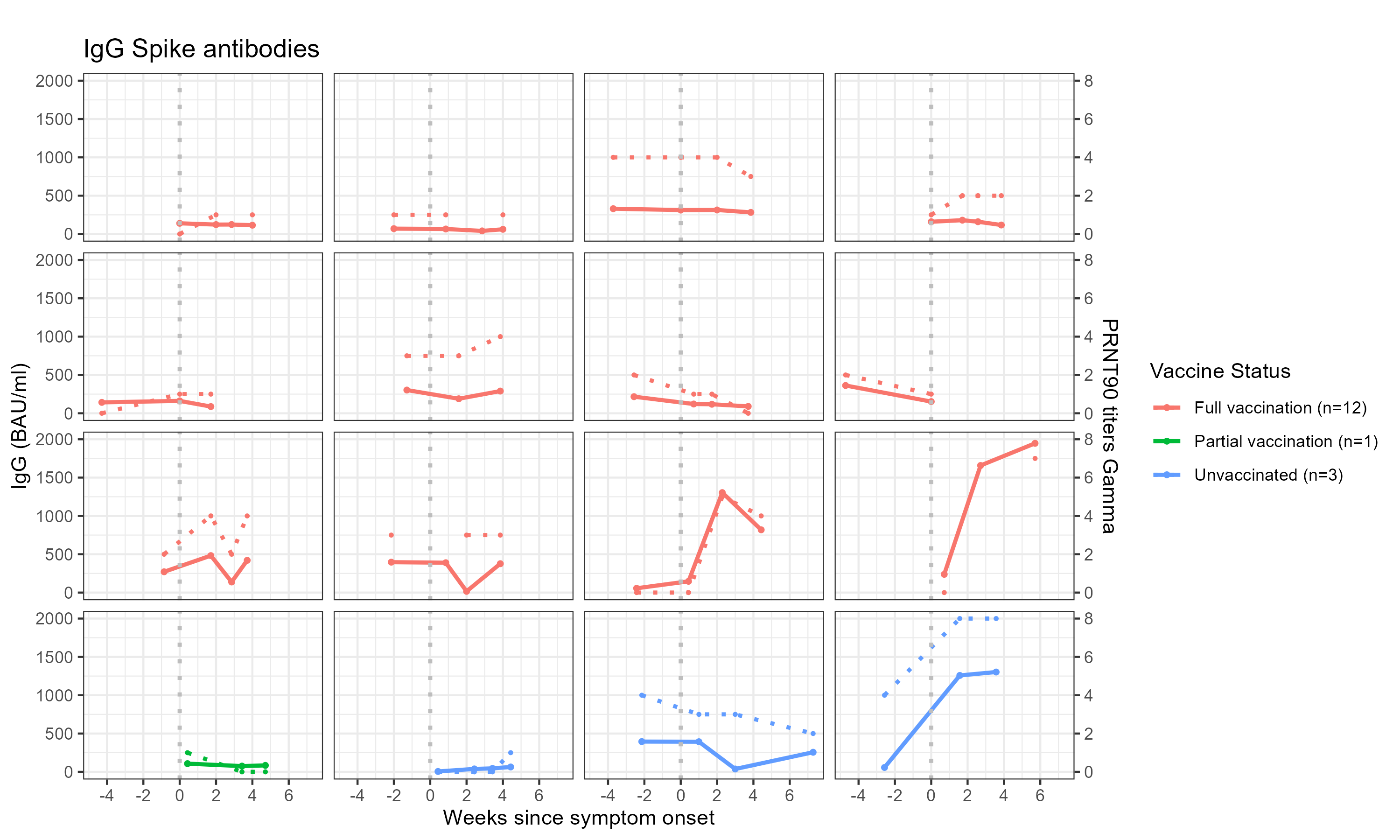

Supplement: ciaf318_Supplementary_Data [file ciaf318_supplementary_data.zip › Figure S5.tiff]
